# Supplementary material for: Modulating Pt-O-Pt atomic clusters with isolated cobalt atoms for enhanced hydrogen evolution catalysis
Source: Nat Commun. 2022 May 4;13:2430. doi: 10.1038/s41467-022-30155-4 (PMC9068789; doi:10.1038/s41467-022-30155-4)
Supplement: Supplementary file 1 — Supplementary Information [file 41467_2022_30155_MOESM1_ESM.pdf]

Supplementary Information

Modulating Pt-O-Pt atomic clusters with isolated cobalt atoms for enhanced hydrogen evolution catalysis

Yufei Zhao[1], Priyank V. Kumar[1], Xin Tan[2], Xinxin Lu[3], Xiaofeng Zhu[1], Junjie Jiang[1], Jian Pan[1], Shibo Xi[4], Hui Ying Yang[5], Zhipeng Ma[1], Tao Wan[6], Dewei Chu[6], Wenjie Jiang[1], Sean C. Smith[2], Rose Amal[1], Zhaojun Han[*,1,7], Xunyu Lu[*,1]

[1] Particles and Catalysis Research Laboratory, School of Chemical Engineering, The University of New South Wales, Sydney, NSW 2052, Australia

[2] Integrated Materials Design Laboratory, Department of Applied Mathematics, Research School of Physics, Australian National University, Canberra, ATC 2601, Australia

[3] School of Science and Engineering, The Chinese University of Hong Kong (Shenzhen), Shenzhen 518172, China

[4] Institute of Chemical & Engineering Sciences, Agency for Science, Technology and Research (A*STAR), 627833, Singapore

[5] Singapore University of Technology and Design, 8 Somapah road, Singapore 487372

[6] School of Materials Science and Engineering, The University of New South Wales, Sydney, NSW 2052, Australia

[7] CSIRO Manufacturing, 36 Bradfield Road, Lindfield, NSW 2070, Australia

Corresponding authors: Xunyu Lu, Zhaojun Han

Email: xunyu.lu@unsw.edu.au; zhaojun.han@unsw.edu.au

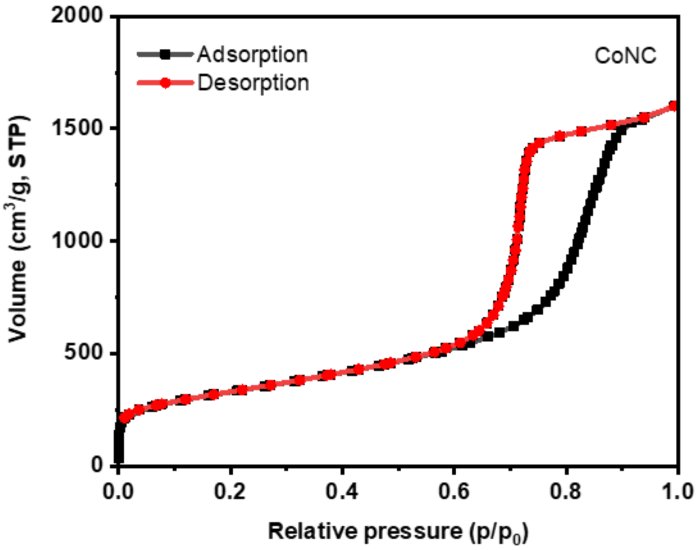

Supplementary Figure 1. Nitrogen adsorption/desorption isotherm of CoNC. The calculated specific surface area in BET method of 1147.4 m^2 g^{-1}.

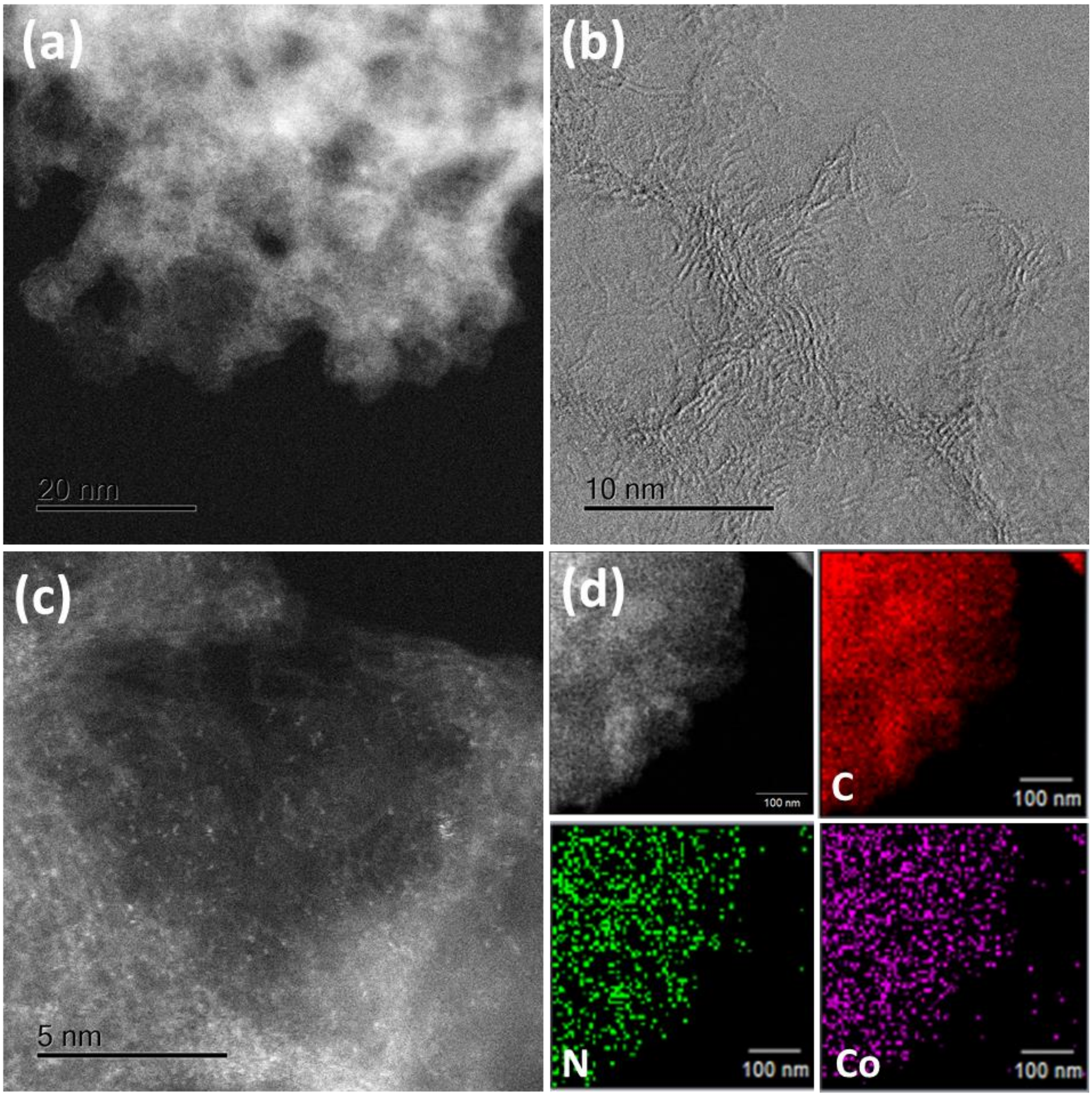

Supplementary Figure 2. Morphological characterization of CoNC. (a-c) HAADF-STEM and high-resolution TEM images of CoNC, (d) HAADF-STEM elemental distribution of the C, N and Co elements in CoNC.

The HAADF-STEM image of CoNC in Supplementary Figure 2a shows that the highly porous structure with partially graphitic properties (HRTEM image in Supplementary Figure 2b). No particles or clusters were observed on the entire carbon matrix. The high-resolution HAADF-STEM image of CoNC in Supplementary Figure 2c displays a large number of bright dots spreading in the porous carbon support, which correspond to the atomically dispersed Co atoms. The elemental mappings in Supplementary Figure 2d reveal the homogeneous distribution of C, N and Co elements on the entire nanostructure.

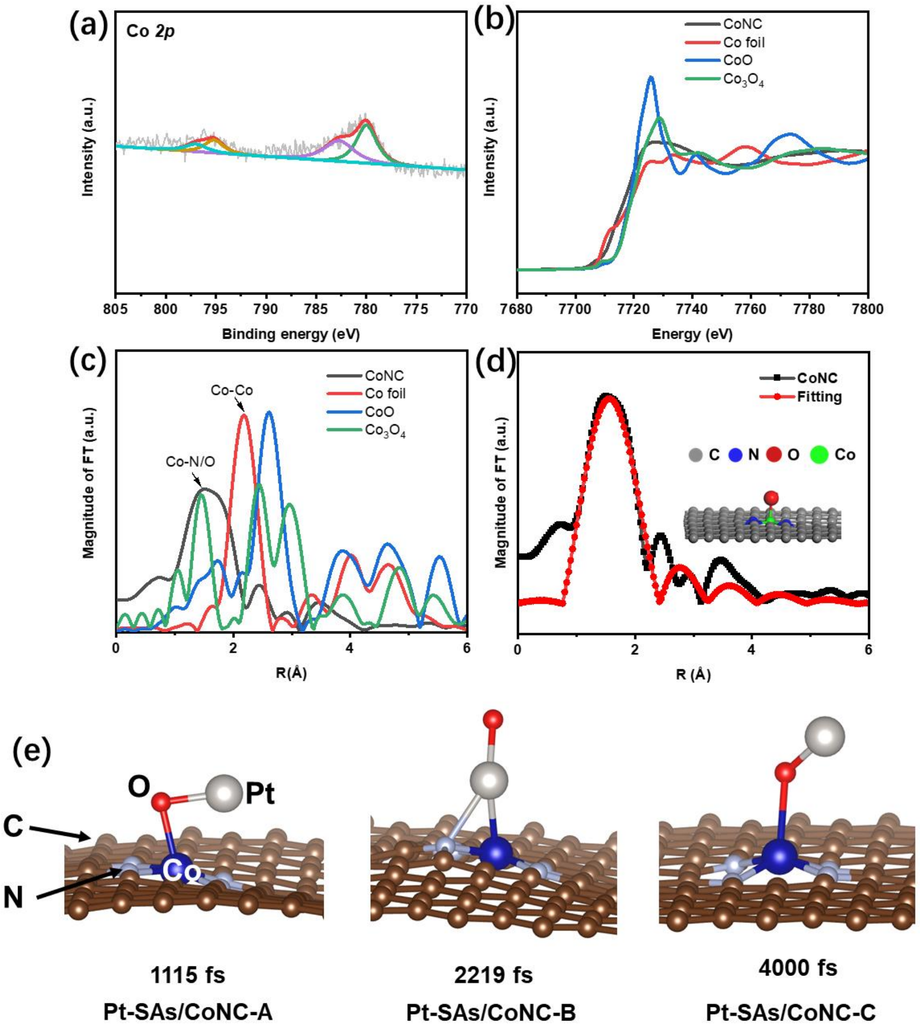

Supplementary Figure 3. The characterization of CoNC. (a) The high-resolution XPS result of Co *2p*, (b-c) Normalized XANES spectra and the k^3-weighted Fourier transform of EXAFS spectra at the Co *k*-edge of Co foil, CoO, CoNC and Co_3O_4. (d) EXAFS curves between the experimental data and the fit of CoNC. (e) Three different metastable configurations of Pt-SAs/CoNC-A, Pt-SAs/CoNC-B and Pt-SAs/CoNC-C.

In order to understand the role of O in facilitating Pt binding to CoNC units, we performed an additional *Ab initio* molecular dynamics (AIMD) simulation by randomly placing O and Pt atoms in the vicinity of the CoNC unit and allowing the system to equilibrate (see DFT methods). Our results reveal three possible metastable configurations of Pt SAs on CoNC, which are shown in Supplementary Figure 3e (named as Pt-SAs/CoNC-A, Pt-SAs/CoNC-B and Pt-SAs/CoNC-C), obtained at different time intervals during the AIMD run. Further DFT relaxations indicate that the Pt-SAs/CoNC-C is the most stable of these three structures. In this configuration, the O atom acts as an anchoring link for Pt species to bind to the CoN_4 unit. Given that this is the most stable configuration, it clearly highlights the critical role played by the O atom as an anchor to capture the incorporated Pt ions forming protrusion atomic structure.

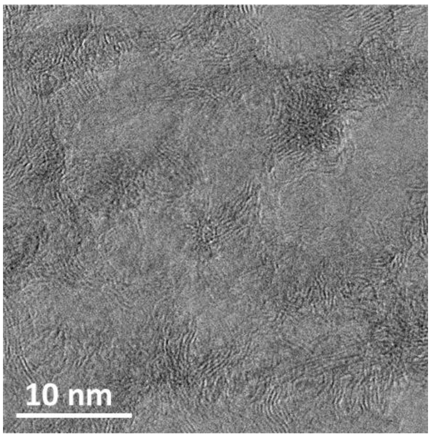

Supplementary Figure 4. Morphological characterization of Pt-ACs/CoNC. TEM image of Pt-ACs/CoNC shows the partially graphitic properties.

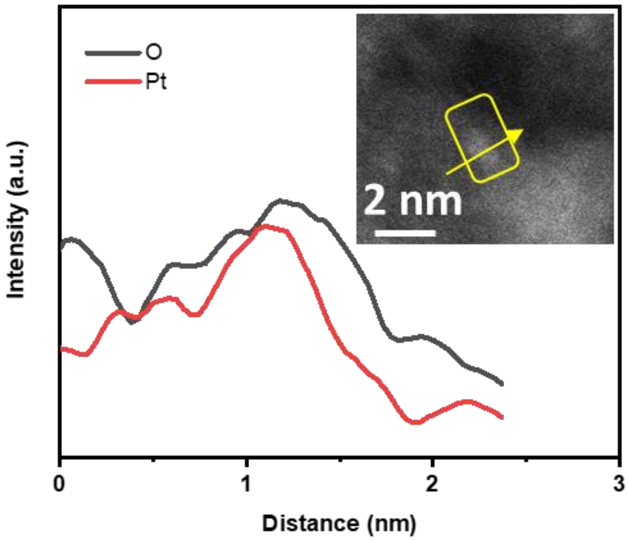

Supplementary Figure 5. EDS analysis of Pt-ACs/CoNC. Result shows a similar trend of Pt and O atoms in one atomic cluster. The inset is the high-resolution HAADF-STEM image of Pt-ACs/CoNC at a low voltage of 60 kV.

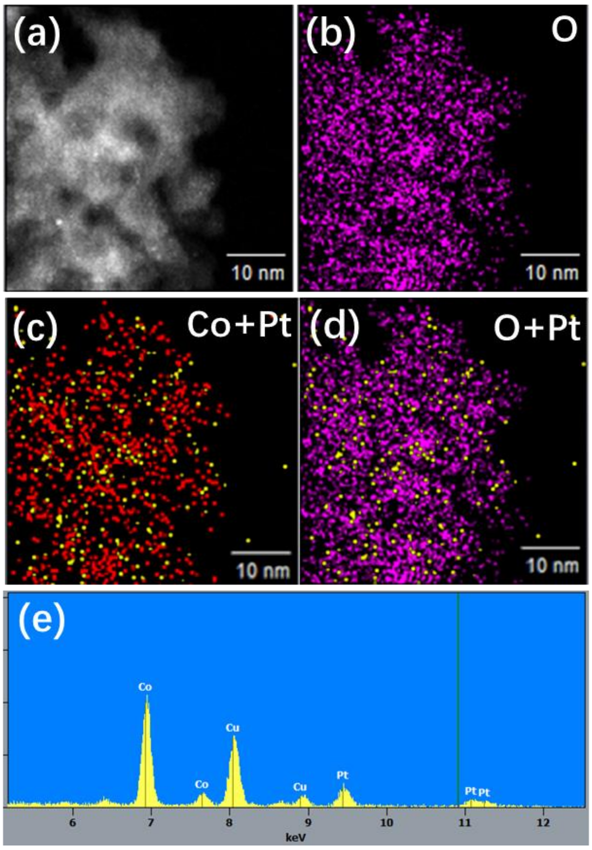

Supplementary Figure 6. EDS analysis of Pt-ACs/CoNC. (a) HAADF-STEM image, and elemental mapping of element O (b), Co+Pt (c), Pt+O (d), (e) EDS spectrum of Co and Pt.

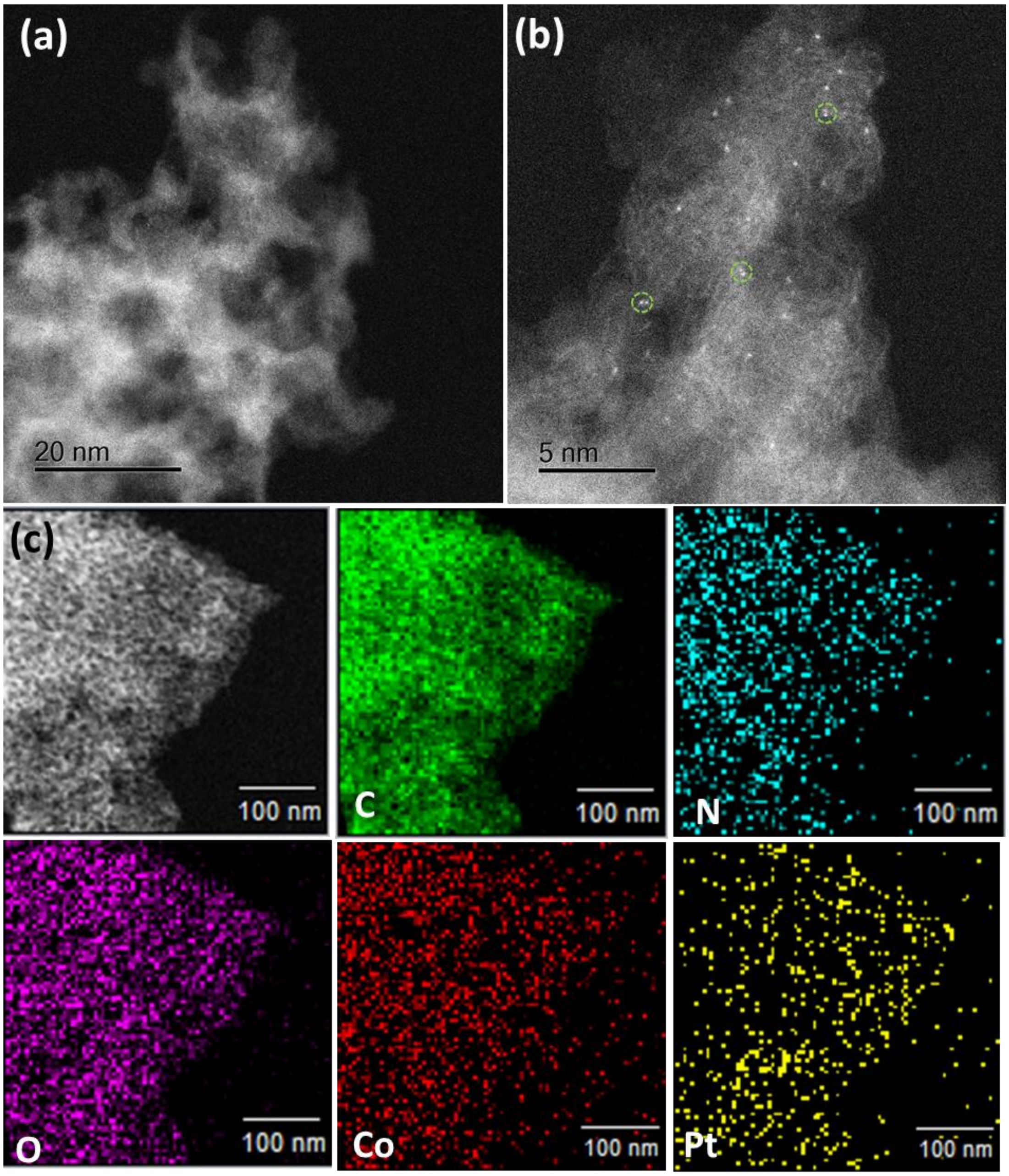

Supplementary Figure 7. Morphological characterization of Pt-SAs/CoNC. (a, b) HAADF-STEM images of Pt-SAs/CoNC, (c) HAADF-STEM elemental distribution of the C, N, O, Co and Pt elements in Pt-SAs/CoNC.

The HAADF-STEM image of Pt-SAs/CoNC in Supplementary Figure 7a shows the highly porous structure and no particles or clusters were observed on the entire carbon matrix. The elemental mappings in Supplementary Figure 7c reveal the homogeneous distribution of C, N, O, Co and Pt elements on the entire nanostructure.

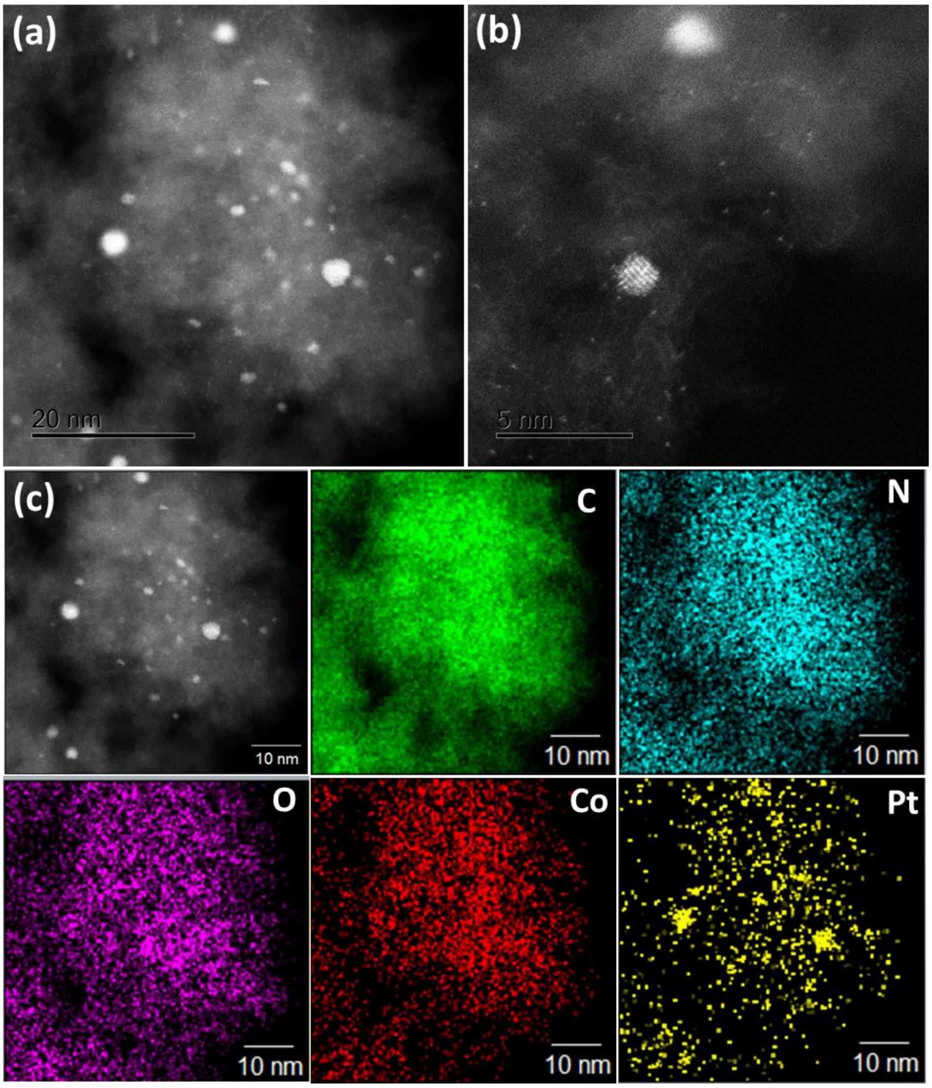

Supplementary Figure 8. Morphological characterization of Pt-NPs/CoNC. (a, b) HAADF-STEM images of Pt-NPs/CoNC, (c) HAADF-STEM elemental distribution of the C, N, O, Co and Pt elements in Pt-NPs/CoNC.

The HAADF-STEM image of Pt-NPs/CoNC in Supplementary Figure 8a-b shows the nanoparticles randomly distributed on the entire CoNC matrix. The elemental mappings in Supplementary Figure 8c reveal the homogeneous distribution of C, N, O and Co elements on the entire nanostructure and confirm the nanoparticles are ascribed to element Pt.

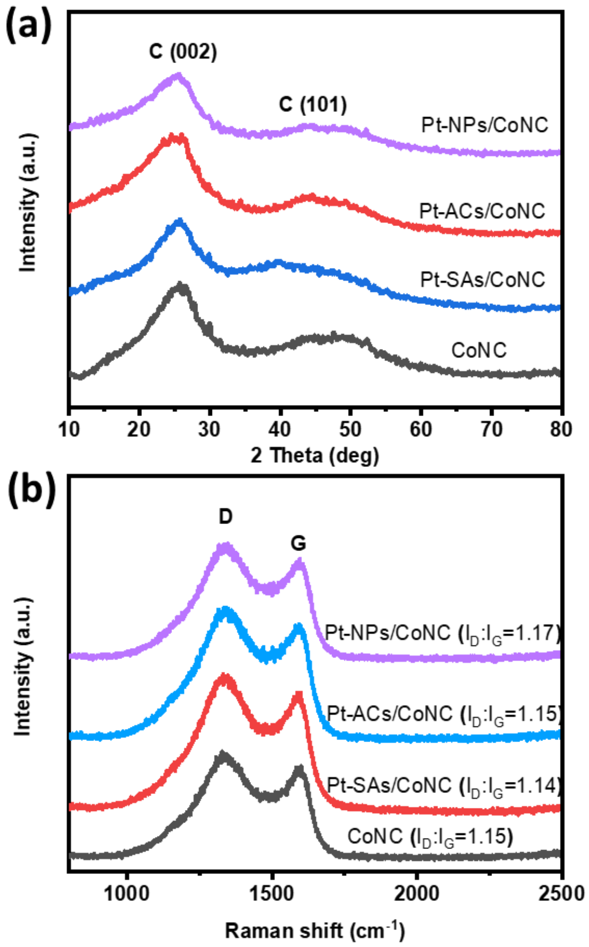

Supplementary Figure 9. Structural characterization of the as-prepared catalysts. (a) the XRD and (b) Raman spectra of CoNC, Pt-SAs/CoNC, Pt-ACs/CoNC, Pt-NPs/CoNC

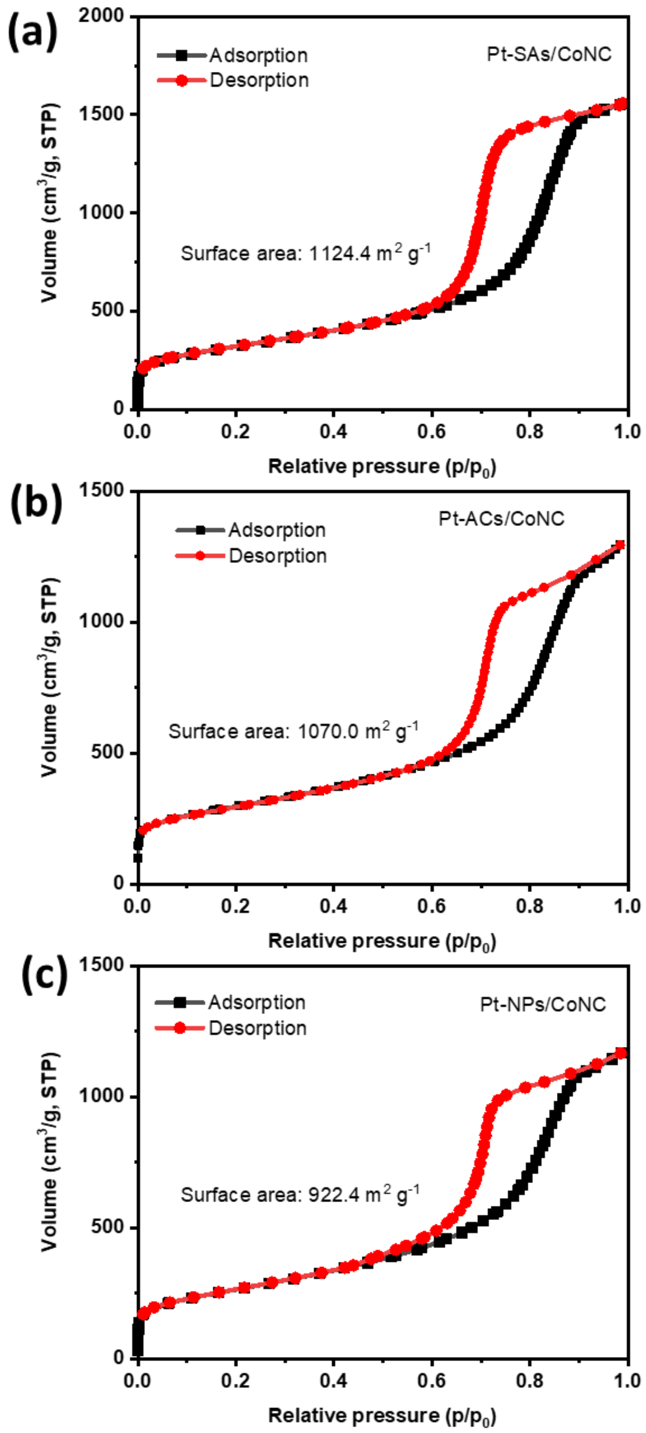

Supplementary Figure 10. Nitrogen adsorption/desorption isotherm of Pt-SAs/CoNC, Pt-ACs/CoNC and Pt-NPs/CoNC. The specific surface areas of (a) Pt-SAs/CoNC, (b) Pt-ACs/CoNC and (c) Pt-NPs/CoNC are 1124.4, 1070.0 and 922.4 m^2 g^{-1}, respectively.

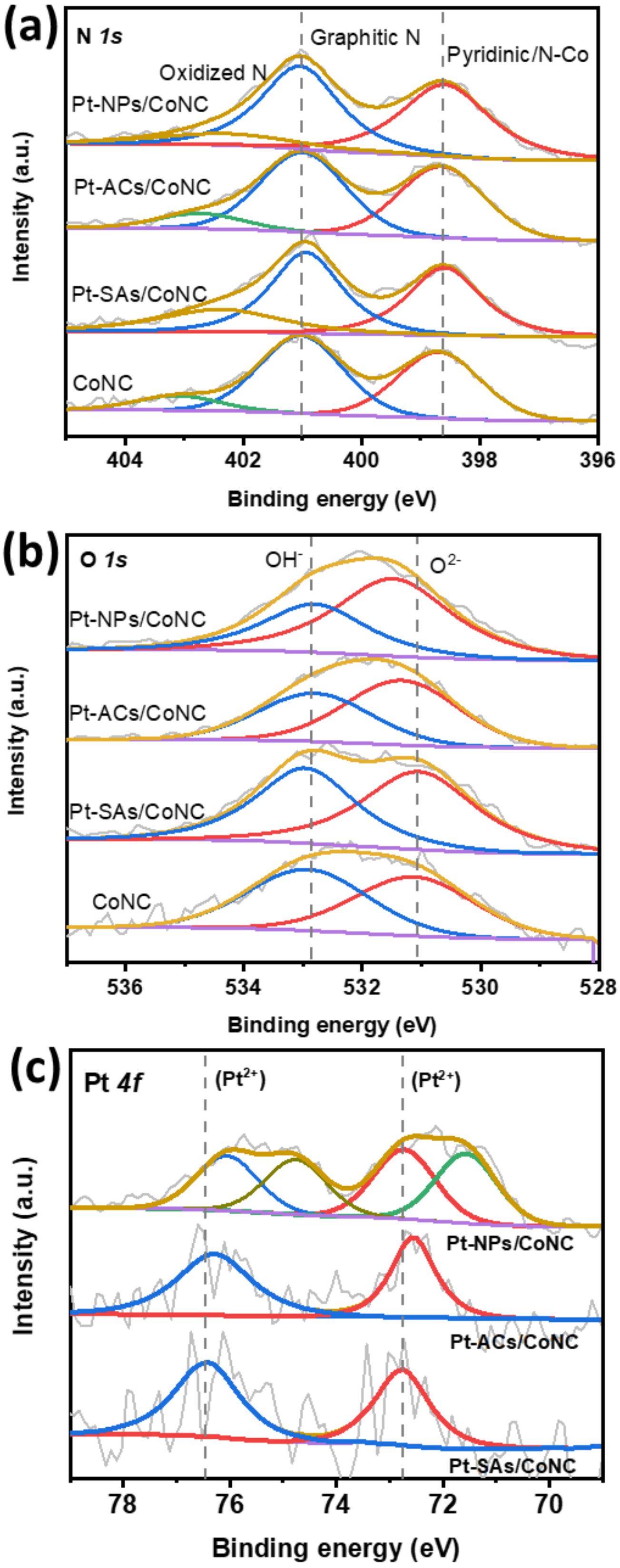

Supplementary Figure 11. The XPS spectra of the as-prepared catalysts. (a) the high resolution of N *1s*, (b) the high resolution of O *1s*, (c) the high resolution of Pt *4f* of CoNC, Pt-SAs/CoNC, Pt-ACs/CoNC and Pt-NPs/CoNC.

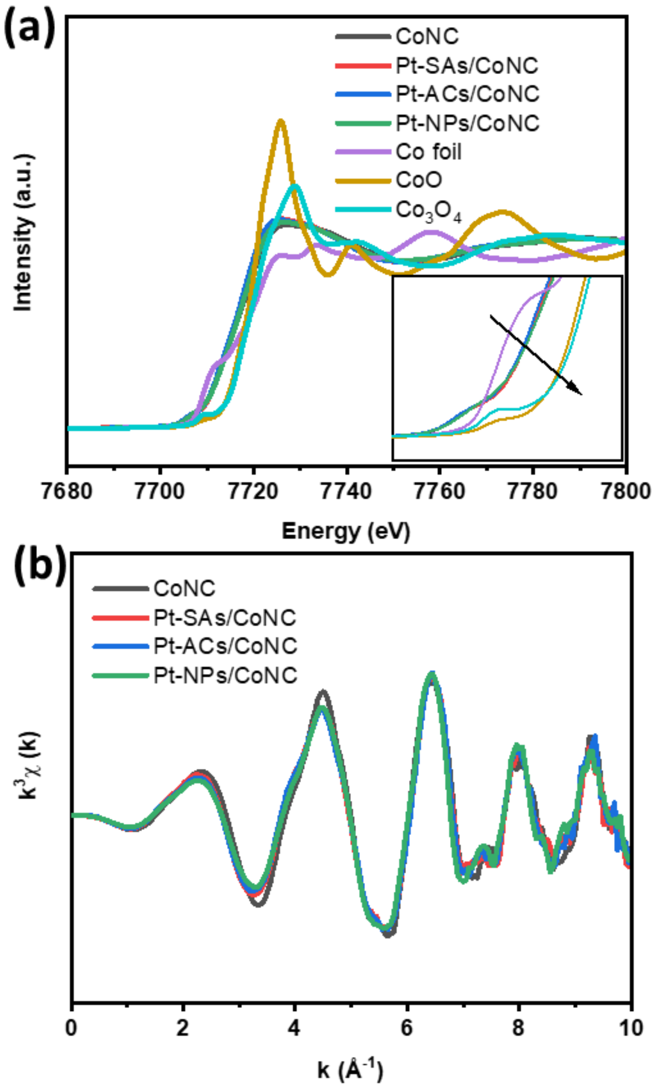

Supplementary Figure 12. Structural characterizations of Pt-ACs/CoNC. The normalized XANES spectra and the k^3-weighted Fourier transform of EXAFS spectra at (a) Co *k*-edge of CoNC, Pt-SAs/CoNC, Pt-ACs/CoNC, Pt-NPs/CoNC and the reference materials. (b) The k^3-weighted EXAFS in *K*-space for CoNC, Pt-SAs/CoNC, Pt-ACs/CoNC, Pt-NPs/CoNC and the reference materials.

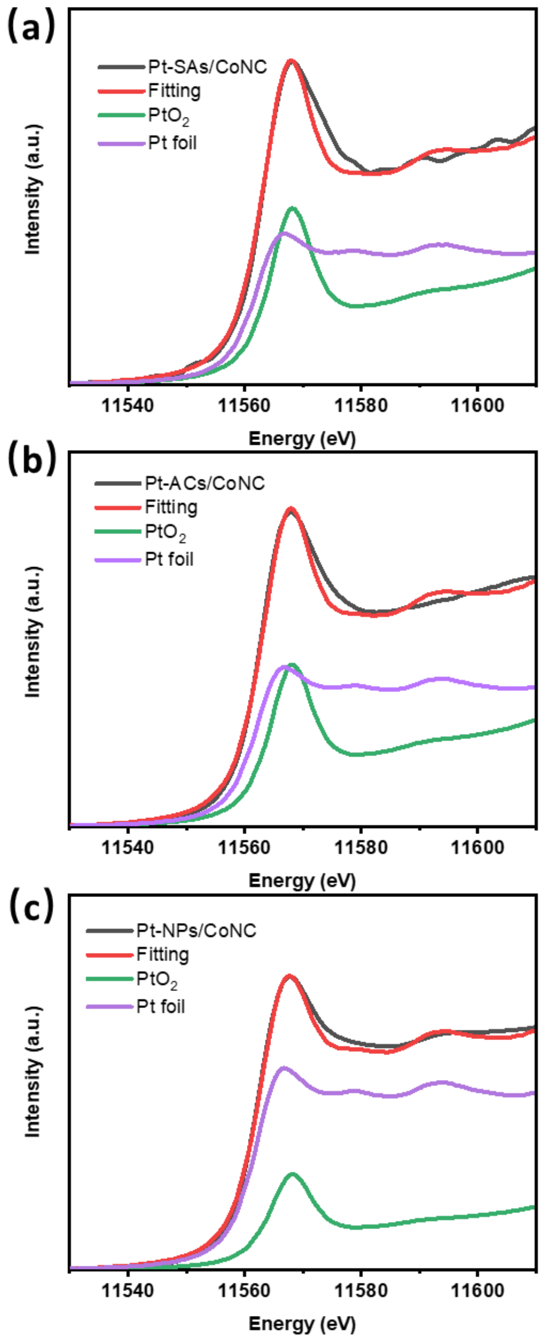

Supplementary Figure 13. The calculation of Pt valence state. The fitting results of (a) Pt-SAs/CoNC, (b) Pt-ACs/CoNC, (c) Pt-NPs/CoNC based on Pt foil and PtO_2 to calculate the oxidation state of Pt species.

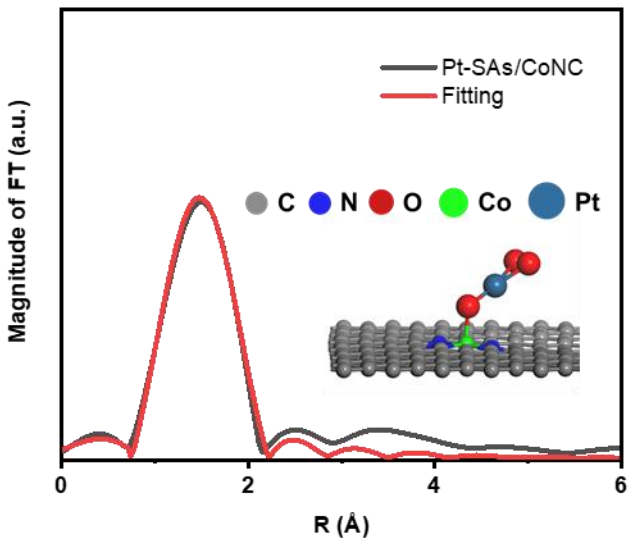

Supplementary Figure 14. The fitting results of Pt-SAs/CoNC. EXAFS curves between the experimental data and the fit of Pt-SAs/CoNC, the inset is the fitted structure.

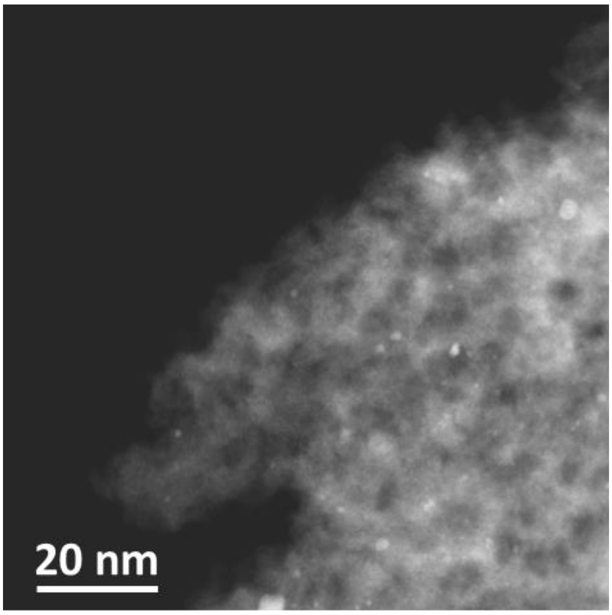

Supplementary Figure 15. Morphological characterization of Pt-NPs/NC. TEM image of Pt-NPs/NC.

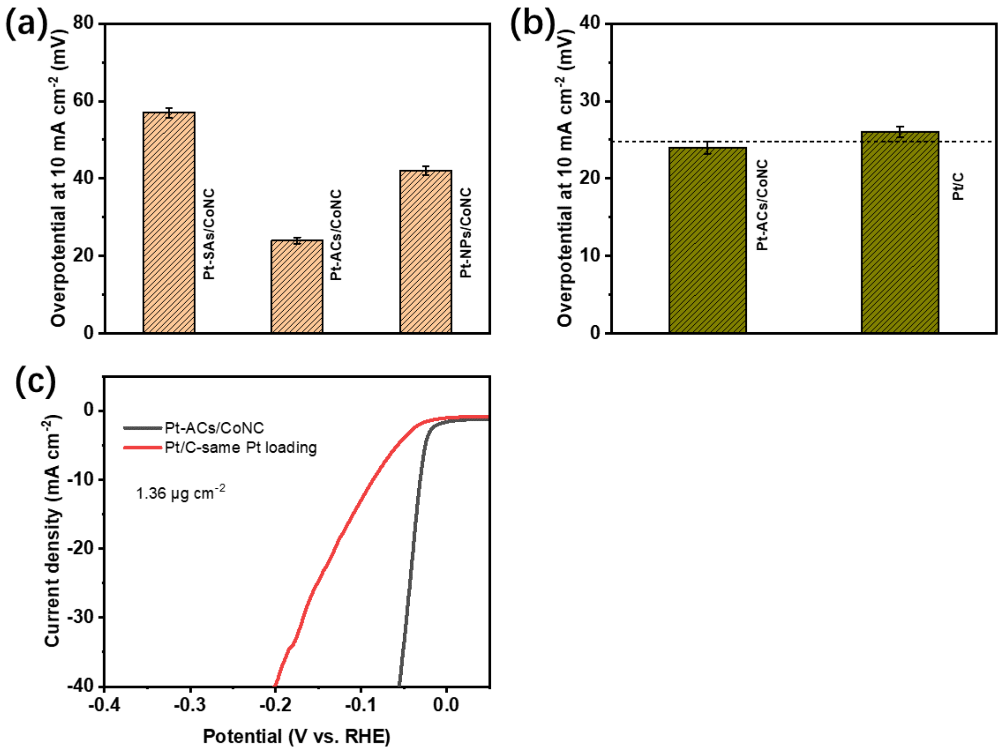

Supplementary Figure 16. The error analysis and LSV results of the as-prepared catalysts and commercial Pt/C. (a) The overpotential of current density of 10 mA cm^{-2} for Pt-SAs/CoNC, Pt-ACs/CoNC and Pt-NPs/CoNC with the error analysis. (b) the overpotential of current density of 10 mA cm^{-2} for Pt-ACs/CoNC and commercial Pt/C with the error analysis. (c) The electrochemical performance test of Pt-ACs/CoNC and commercial Pt/C with same Pt loading on the working electrode (different total loading).

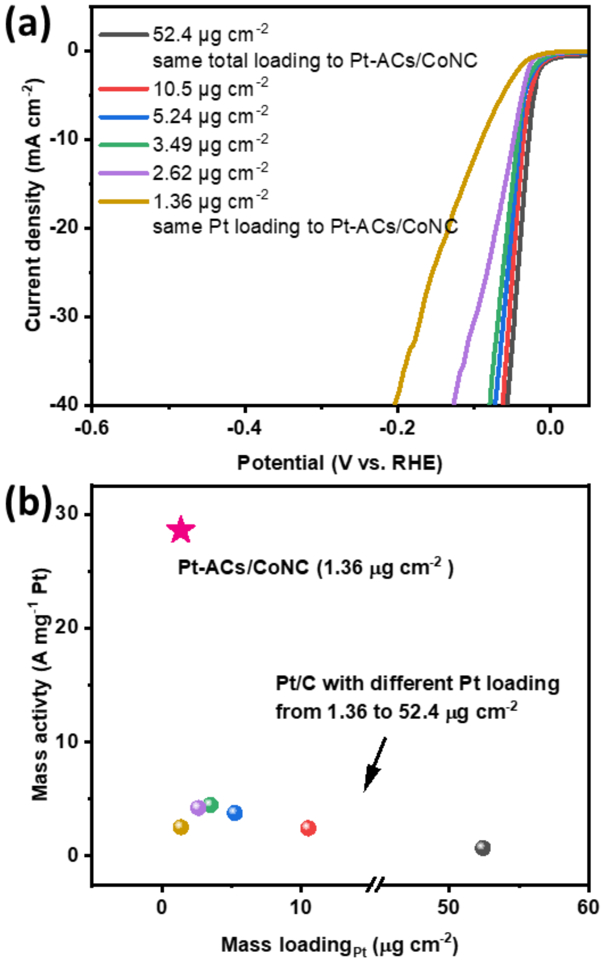

Supplementary Figure 17. The electrochemical performance test of commercial Pt/C with different Pt loading on the working electrode with same geometric surface area. (a) LSV results and (b) the corresponding mass activity of commercial Pt/C.

We have tested the electrochemical performance of commercial Pt/C with different element Pt loading. As shown in Figure Supplementary Figure 17a, it can be observed that the HER activity of Pt/C only decreases slightly when the Pt loading is lowered from 52.4 to 3.49 μg cm^{-2}, thereby leading to the increased mass activity. As a result, at the applied overpotential of 50 mV, the highest mass activity of Pt/C, 4.48 A mg^{-1}_{Pt}, is obtained with the Pt loading of 3.49 μg cm^{-2}. Further reducing the Pt loading will lead to a rapid drop in the HER activity, therefore lowering the mass activity of Pt/C. As shown in Supplementary Figure 17, the mass activity of Pt/C with 1.36 μg cm^{-2} Pt loading is only 2.53 A mg^{-1}_{Pt} at the applied overpotential of 50 mV. The mass activity for the as-prepared Pt-ACs/CoNC with Pt mass loading of 1.36 μg cm^{-2} is 28.6 A mg^{-1}_{Pt} at the overpotential of 50 mV, which is at least more than 6 times higher than the Pt/C catalysts with any Pt loadings, demonstrating the excellent intrinsic catalytic activity of Pt-ACs/CoNC for HER.

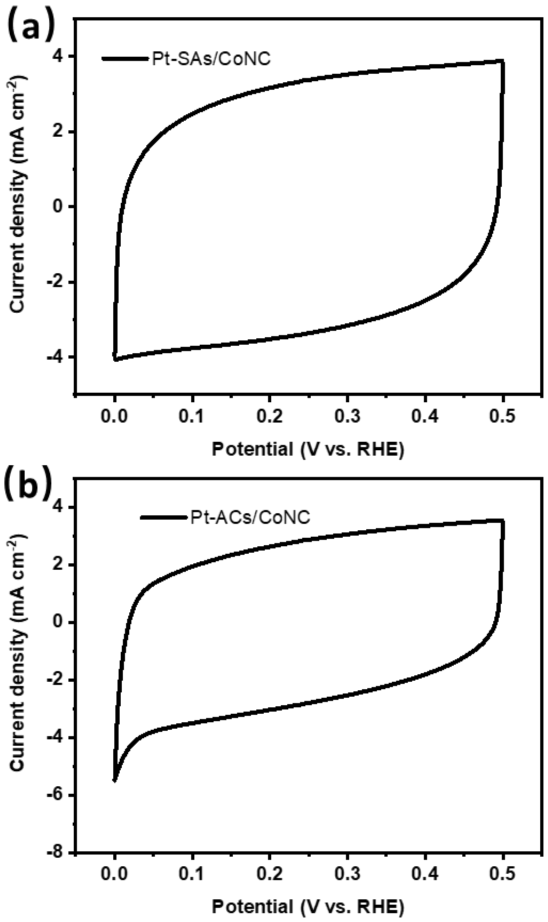

Supplementary Figure 18. Cyclic voltammograms measurements. (a) Pt-SAs/CoNC and (b) Pt-ACs/CoNC in N_2-saturated 0.5 M H_2SO_4 solution with a scan rate of 50 mV s^{-1}.

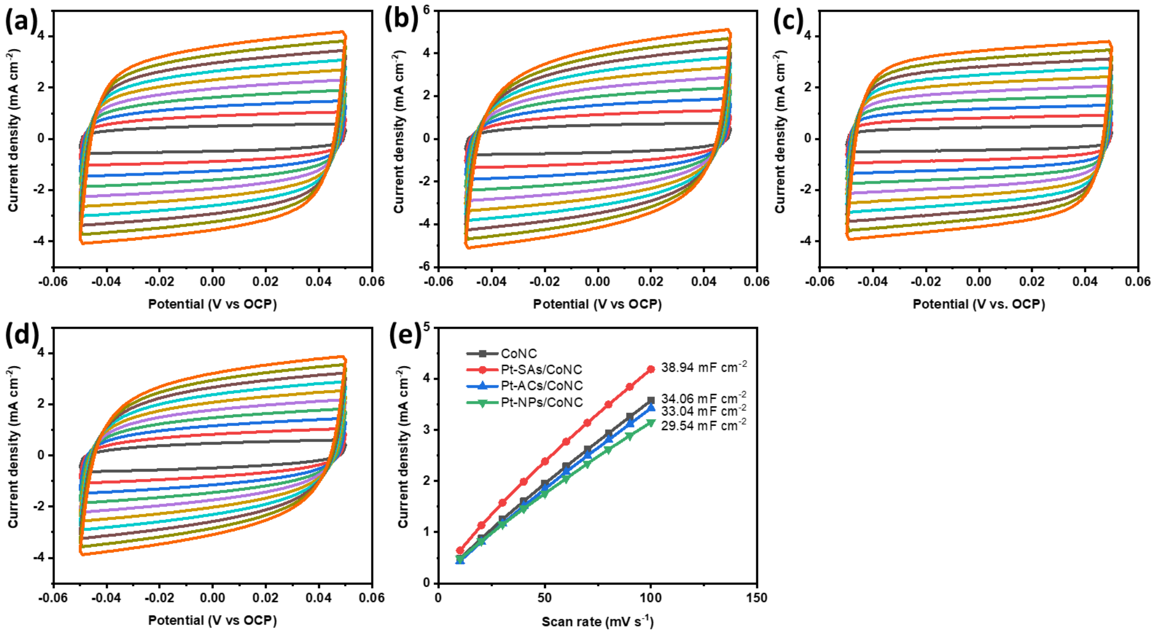

Supplementary Figure 19. The calculations of electrochemically active surface area of the as-prepared catalysts. Cyclic voltammograms are performed in 0.5 M H_2SO_4 solution in a potential window without faradaic processes, (a) CoNC, (b) Pt-SAs/CoNC, (c) Pt-ACs/CoNC and (d) Pt-NPs/CoNC. (e) Scan rate dependence of the average capacitive currents for CoNC, Pt-SAs/CoNC, Pt-ACs/CoNC and Pt-NPs/CoNC.

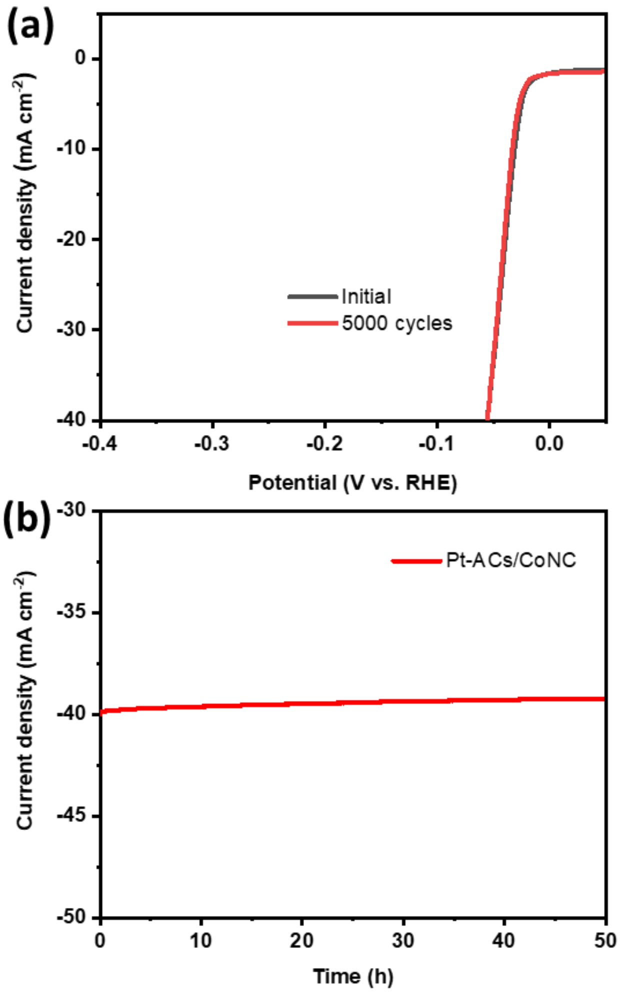

Supplementary Figure 20. Stability test of Pt-ACs/CoNC. (a) LSV cycling for 5000 cycles at an accelerated scan rate of 100 mV s^{-1} with the potential range between 0.10 and -0.15 V *vs*. RHE, (b) Chronoamperometry curve to achieve the current density of 40 mA cm^{-2} at a static overpotential of 63 mV *vs*. RHE.

The long-term stability tests for Pt-ACs/CoNC catalyst show extremely high stability with no decay in HER performance for 5000 cycles and slight degradation at the current density of 40 mA cm^{-2} for 50 h (2.0 % degradation).

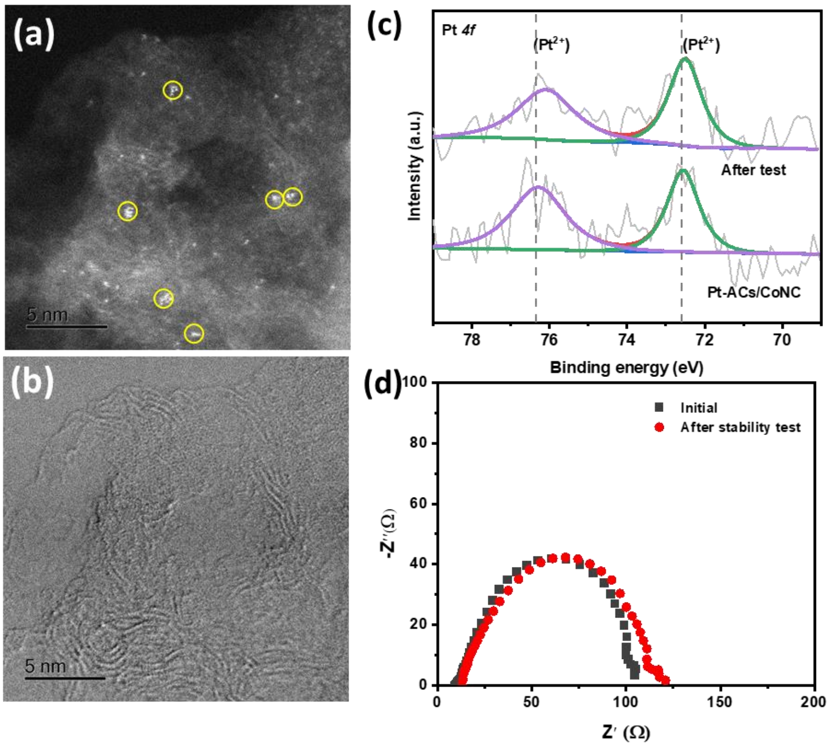

Supplementary Figure 21. The characterization of Pt-ACs/CoNC after the stability test. (a-b) HAADF-STEM images of Pt-ACs/CoNC after the stability test, show the atomic clusters are well maintained after the long-term HER process (the yellow circled area). (c) the high resolution of Pt *4f* before and after the stability test. (d) EIS results before and after the stability test.

The HAADF-STEM and high-resolution TEM images in Supplementary Figure 21a-b show the highly porous and graphitic structure has been well retained. Besides, the Pt ACs are also well maintained after the long-term HER process without observing any nanoparticles. The high-resolution XPS spectra of Pt *4f* in Supplementary Figure 21c show the Pt species after the stability test still possess cationic nature, further indicating the Pt ACs are well maintained. Moreover, the slightly negative shifted Pt *4f* peaks after the stability test suggest the Pt species are slightly reduced during HER process, which is beneficial to hydrogen generation. The EIS results in Supplementary Figure 21d show the spectra before and after the stability test remain almost unchanged, suggesting that Pt-ACs/CoNC is not altered during the long-term stability HER test.

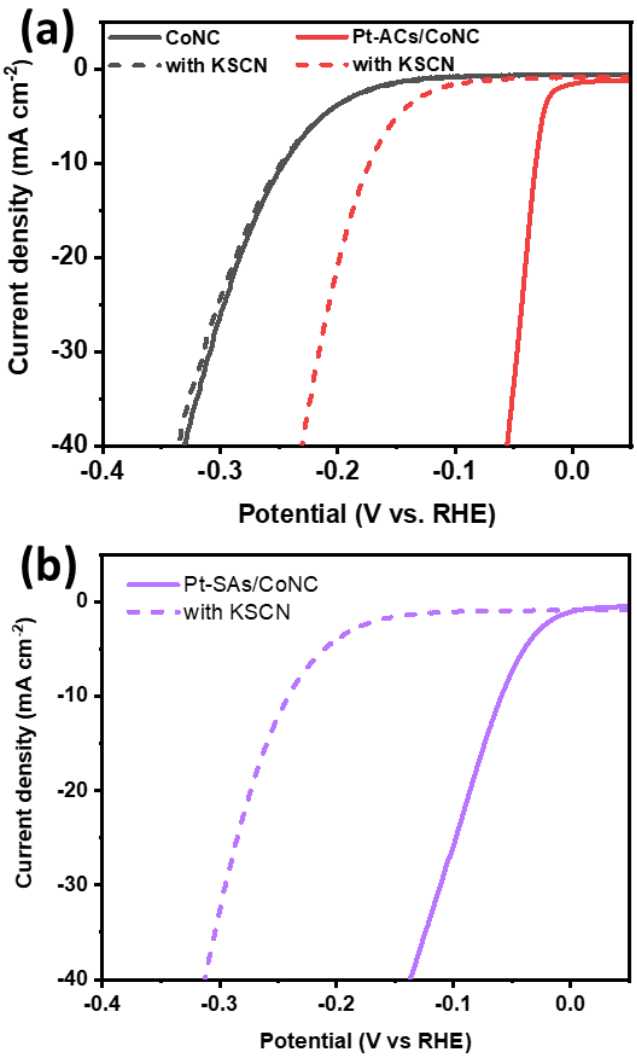

Supplementary Figure 22. The influence of thiocyanate ions on the HER electrocatalytic performance. The LSV results of (a) CoNC and Pt-ACs/CoNC, (b) Pt-SAs/CoNC.

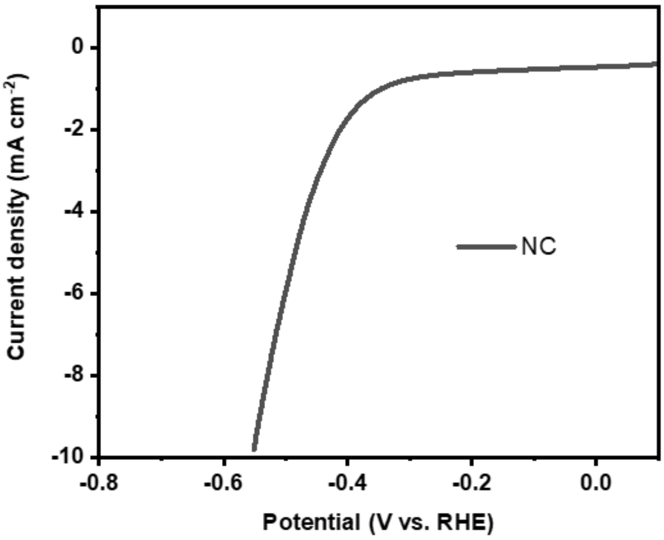

Supplementary Figure 23. HER performance of NC. The polarization curves of NC in 0.5 M H_2SO_4.

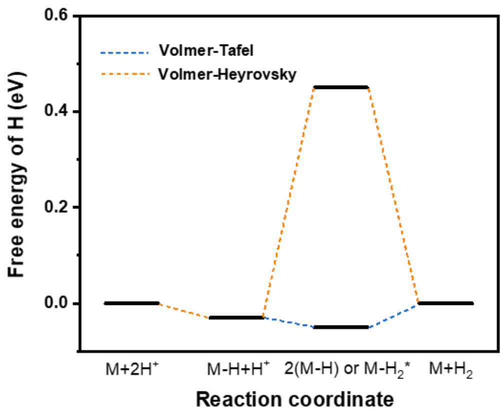

Supplementary Figure 24. Free energy diagrams of Pt-ACs/CoNC. The Volmer-Heyrovsky and Volmer-Tafel catalytic pathways on Pt-ACs/CoNC.

We have explored the HER catalytic mechanism of Pt-ACs/CoNC by using DFT calculations (Volmer-Heyrovsky or Volmer-Tafel). In Volmer-Tafel pathway, two absorbed hydrogen atoms on active sites combine to give H_2. In contrast, in the Volmer-Heyrovsky pathway, the transfer of a second electron to the absorbed hydrogen atom is coupled to the transfer of another proton from the solution to evolve H_2. We have modelled both these pathways using DFT and the free energy diagram is shown in Supplementary Figure 24. The results show that the Volmer-Tafel pathway is more favourable since the reaction energy toward H_2 evolution is lower. Thus, Volmer-Tafel pathway is the dominant HER catalytic process of Pt-ACs/CoNC, which is consistent with our experimental findings. Instead, we find that there is relatively larger reaction energy for H_2 molecule to form at a Pt site of Pt-ACs/CoNC *via* the Volmer-Heyrovsky pathway, thus making it less probable in our experiments. Therefore, both of the DFT calculations and experimental results demonstrate the Volmer-Tafel pathway is more favourable for Pt-ACs/CoNC in HER process.

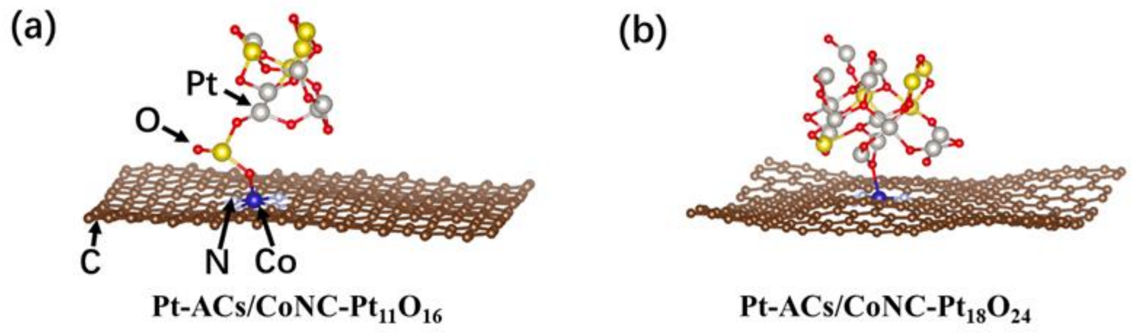

Supplementary Figure 25. The atomic structure of Pt-ACs/CoNC. (a) $Pt_{11}O_{16}$ and (b) $Pt_{18}O_{24}$ supported by Co single atoms.

In addition to the atomic structure we have constructed in Figure 5 in the manuscript, we have proposed additional computational models of Pt-ACs/CoNC by varying the dimensions of Pt ACs and numbers of Co SAs to better illustrate our findings. To further avoid being too particular, we have constructed $Pt_{11}O_{16}$ and $Pt_{18}O_{24}$ (Pt bonding to O atoms with three coordination numbers) on top of one single Co atom as the anchoring site. After relaxing these configurations by AIMD, the most stable structural configurations obtained are shown in Supplementary Figure 25a-b. We find that the Pt-O-Pt atomic clusters are clearly supported by Co single atoms. Additionally, our simulations reveal that the Pt ACs are bonded to the Co atom *via* an O atom in each case, further demonstrating that O is essential for the attachment of the Pt ACs to the Co SAs. We have further investigated the HER catalytic ability of the aforementioned atomic structures of Pt-ACs/CoNC. We randomly chose five Pt sites (marked as yellow spheres) per structure and evaluated ΔG_H for the H adsorption. The results show the best optimized free energies are -0.03 eV and -0.18 eV for the Pt atoms in $Pt_{11}O_{16}$ and $Pt_{18}O_{24}$, respectively, indicating that they are potentially HER active. Therefore, changing the numbers of Pt and Co atoms in Pt-ACs/CoNC (DFT calculations) can all deliver excellent HER catalytic activity, suggesting the advantages of Pt ACs for HER process.

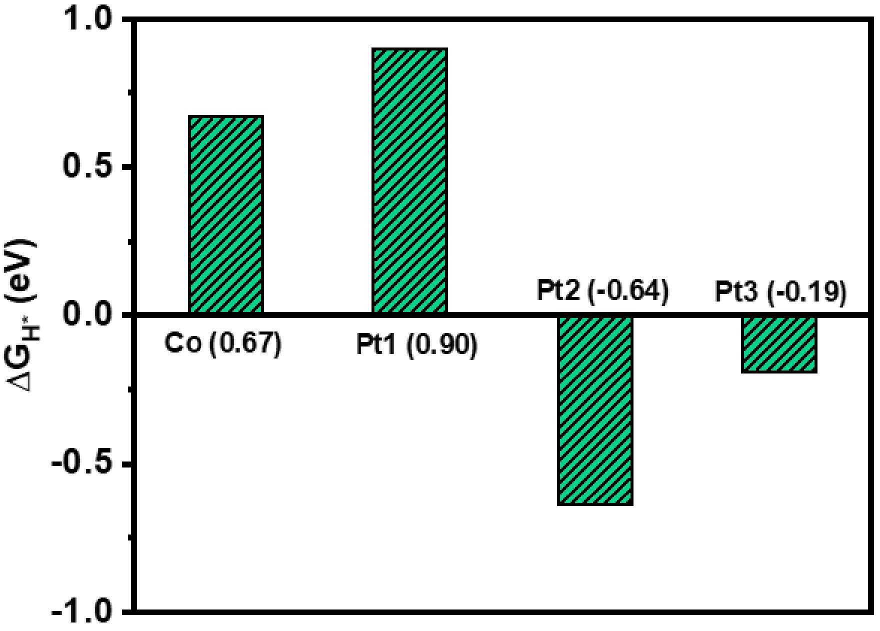

Supplementary Figure 26. Calculated free energy diagram of Pt-ACs/CoNC. Co and Pt sites in Pt-ACs/CoNC (the atomic structure is in Figure 4a).

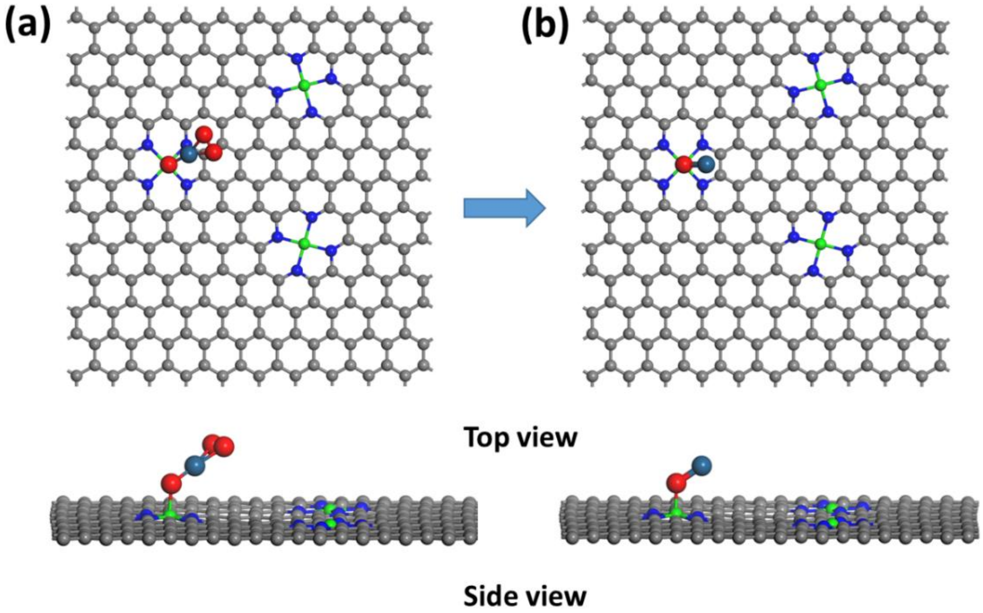

Supplementary Figure 27. The atomic structure of Pt-SAs/CoNC. Pt-SAs/CoNC underwent atomic structure change from (a) to (b) during the HER process.

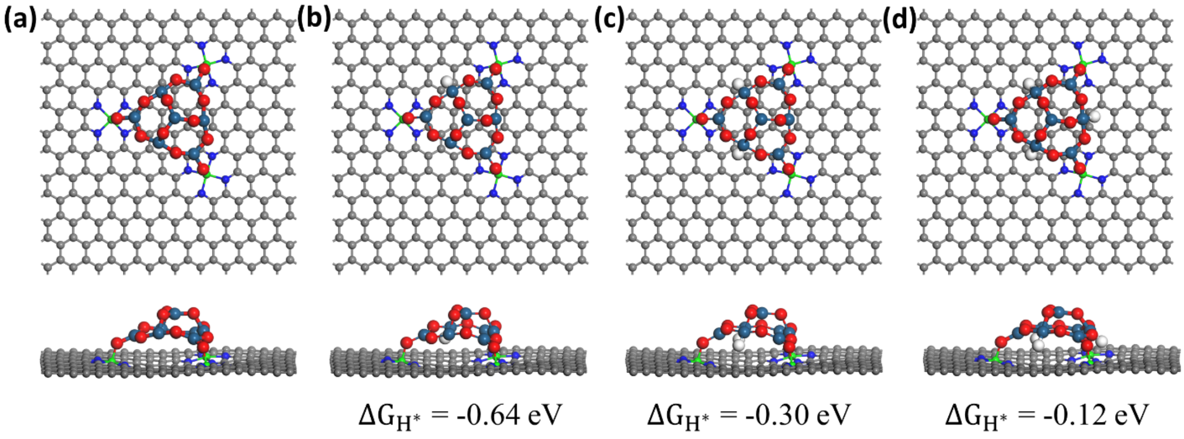

Supplementary Figure 28. Calculated free energy diagram of Pt-ACs/CoNC. Pt atoms in Pt-O-Pt unit with different H* coverage (a) 0 H*, (b) 1 H*, (c) 2 H*, (d) 3 H*.

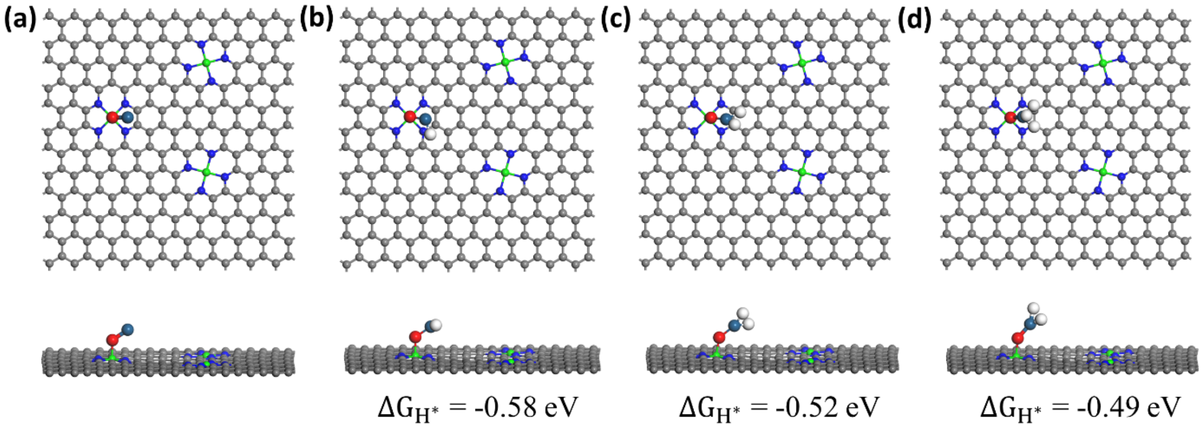

Supplementary Figure 29. Calculated free energy diagram of Pt-SAs/CoNC. Pt atoms with different H* coverage (a) 0 H*, (b) 1 H*, (c) 2 H*, (d) 3 H*.

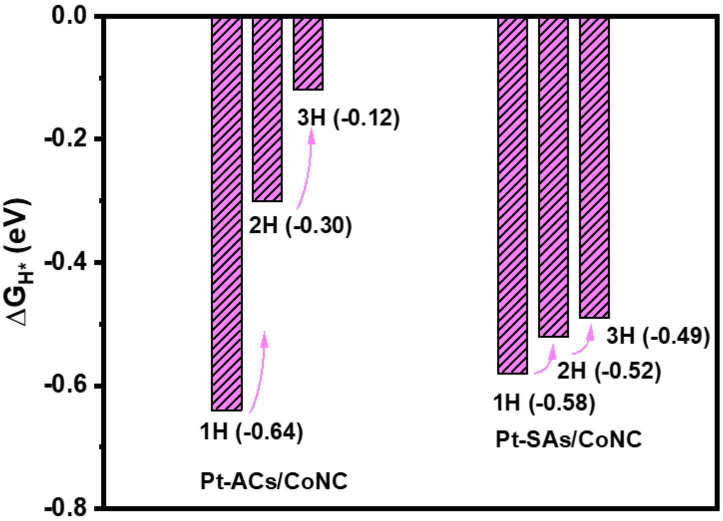

Supplementary Figure 30. Calculated free energy diagram of Pt-ACs/CoNC and Pt-SAs/CoNC. Calculated free energy diagram of Pt atoms in Pt-ACs/CoNC and Pt-SAs/CoNC with different H coverage.

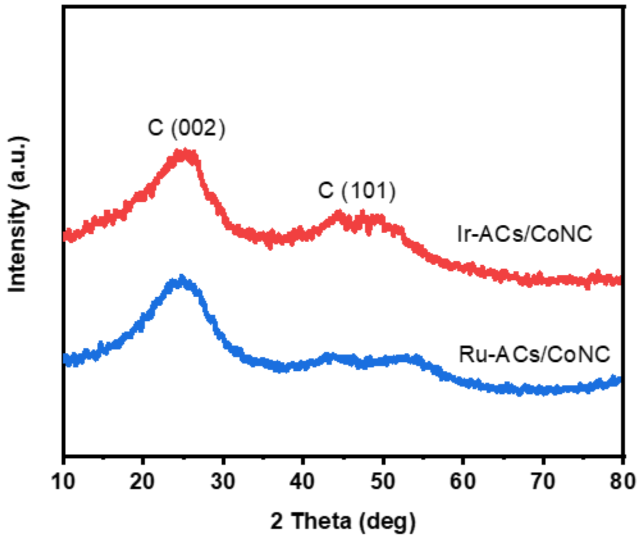

Supplementary Figure 31. Structural characterization of Ru-ACs/CoNC and Ir-ACs/CoNC. The XRD patterns of Ru-ACs/CoNC and Ir-ACs/CoNC.

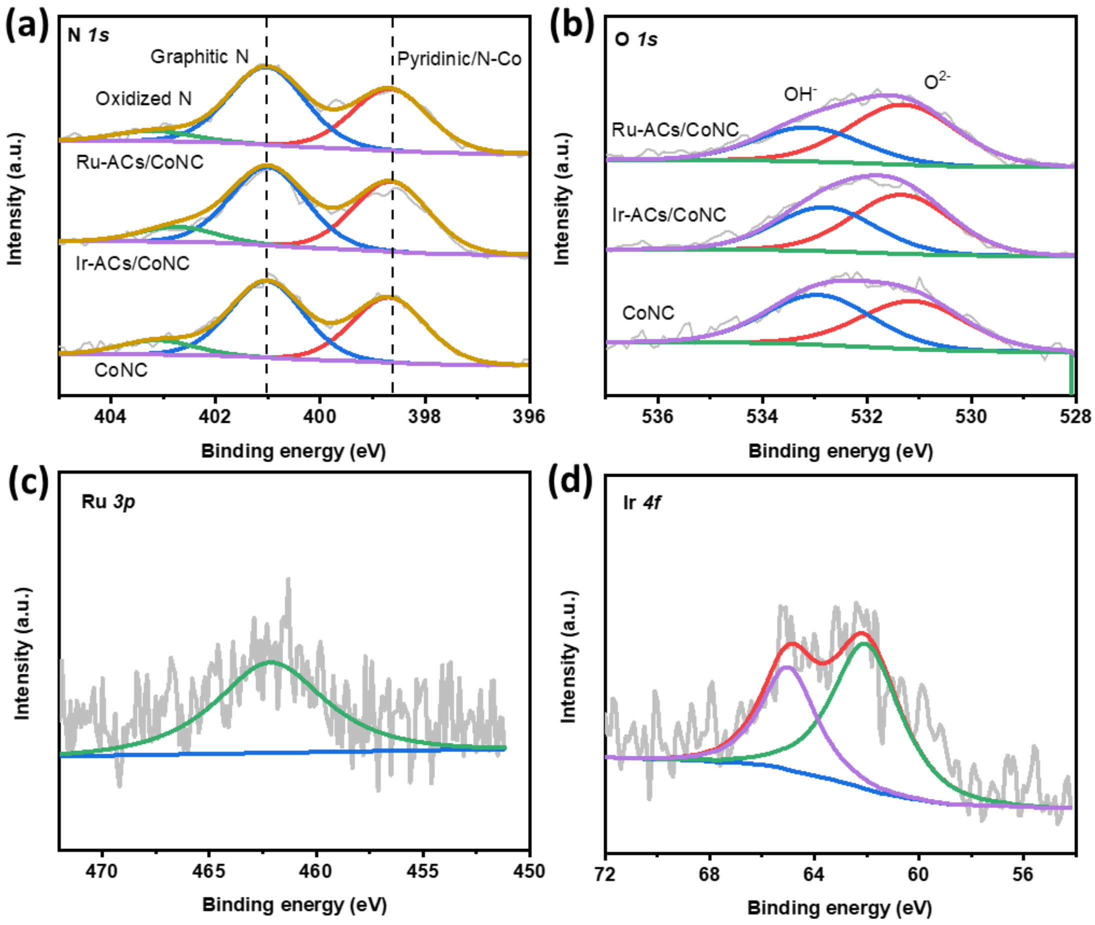

Supplementary Figure 32. The high-resolution XPS spectra of Ru-ACs/CoNC and Ir-ACs/CoNC. (a) N *1s*, (b) O *1s*, (c) Ru *3p* and (d) Ir *4f*.

The high-resolution XPS spectra of N *1s* for Ru-ACs/CoNC and Ir-ACs/CoNC in Supplementary Figure 32a show similar peak positions and intensities to CoNC, which can be deconvoluted into three peaks at 398.64, 401.00, 403.00 eV, corresponding to pyridinic-N/metal-N, graphitic N, and oxidized N, respectively. Similarly, O *1s* spectra of CoNC, Ru-ACs/CoNC and Ir-ACs/CoNC in Supplementary Figure 32b both exhibit peaks at the positions around 532.9 and 531.1 eV, attributing to OH^- and O^{2-}, respectively. The intensities of these peaks, however, change significantly from CoNC to Ru-ACs/CoNC and Ir-ACs/CoNC. The intensity of the peak belonging to O^{2-} increases for Ru-ACs/CoNC and Ir-ACs/CoNC, whereas the intensity of the OH^- peak decreases correspondingly, attributing to the formed Ru/Ir-O bonds in Ru-ACs/CoNC and Ir-ACs/CoNC. The high-resolution XPS of Ru *3p* and Ir *4f* in Supplementary Figure 32c-d reveal the cationic properties of Ru and Ir species in Ru-ACs/CoNC and Ir-ACs/CoNC.

Supplementary Table 1 Fitting result of FT-EXAFS curves shown in Figure 2 and Supplementary Figure 14.

	**Bond type**	**N**	**R(Å)**	**σ^2×10^{-3}(Å^2)**
**Co atom in Pt-ACs/CoNC**	Co-N<br>Co-O	4.72520638	1.98499	0.00300
**Pt atom in Pt-ACs/CoNC**	Pt-O	3.39357002	2.00449	0.00465
**Pt atom in Pt-SAs/CoNC**	Pt-O	3.08413011	1.93362	0.00757

Supplementary Table 2 Comparison of HER activities of different Pt-based materials in 0.5 M H_2SO_4

Entry	Catalysts	Working electrode	Electrolytes	Mass activity at 50 mV (A mg^{-1} Pt)	Areal loading total (Pt) μg cm^{-2}	Overpotential for 10 mA cm^{-2}	Tafel slope (mV dec^{-1})	Reference
**1**	Pt-ACs/CoNC	Glassy carbon	0.5 M H_2SO_4	28.6	262 (1.36)	24	27.7	This work
**2**	Pt-SAs/CoNC	Glassy carbon	0.5 M H_2SO_4	18.8	262 (0.45)	57	64.5	This work
**3**	Pt-NPs/CoNC	Glassy carbon	0.5 M H_2SO_4	4.9	262 (3.85)	42	35.1	This work
**4**	$Mo_2TiC_2T_x$-Pt_{SA}	Carbon paper	0.5 M H_2SO_4	3.07	1000 (12)	30	30	1
**5**	Pt_1/OLC	glassy carbon disk	0.5 M H_2SO_4	~17.76	510 (1.377)	38	35	2
**6**	Pt-AC/DG-150	Glassy carbon	0.1 M $HClO_4$	15.55	100 (1.01)	41	37.8	3
**7**	Pt_5/HMCS-5.08%	Glassy carbon	0.5 M H_2SO_4	20 (30 mV)	~12.8 (0.65)	20.7	28.3	4
**8**	Pt@PCM	Glassy carbon	0.5 M H_2SO_4	2.4	~1300 (6.9)	105	73.6	5
**9**	PtO_x/TiO_2	Glassy carbon slice	0.5 M H_2SO_4	8.68	~65 (0.9)	~150	40	6
**10**	Pt SASs/AG	Glass carbon	0.5 M H_2SO_4	22.4	7068 (31.1)	12	29.33	7
**11**	PtNC/S-C	Glassy carbon	0.5 M H_2SO_4	26.1 (20 mV)	51 (2.55)	11	23.51	8
**12**	PtW_6O_{24}/C	Glassy carbon	0.5 M H_2SO_4	~12.105	/	22	29.8	9
**13**	ALD50Pt/NGNs	Glassy carbon	0.5 M H_2SO_4	10.1	76.5 (1.6)	~38	29	10
**14**	Pt-GDY2	Glassy carbon	0.5 M H_2SO_4	~5.139	/	~65	46.6	11
**15**	Pt1/NMC	Glassy carbon	0.5 M H_2SO_4	9	204 (10)	~30	25	12

References

1. Zhang, J. *et al.* Single platinum atoms immobilized on an MXene as an efficient catalyst for the hydrogen evolution reaction. *Nat. Catal.* **1**, 985-992 (2018).
2. Liu, D. *et al.* Atomically dispersed platinum supported on curved carbon supports for efficient electrocatalytic hydrogen evolution. *Nat. Energy* **4**, 512-518 (2019).
3. Cheng, Q. *et al.* Carbon-defect-driven electroless deposition of Pt atomic clusters for highly efficient hydrogen evolution. *J. Am. Chem. Soc.* **142**, 5594-5601 (2020).
4. Wan, X. K., Wu, H. B., Guan, B. Y., Luan, D. & Lou, X. W. Confining sub-nanometer Pt clusters in hollow mesoporous carbon spheres for boosting hydrogen evolution activity. *Adv. Mater.* **32**, 1901349 (2020).
5. Zhang, H. *et al.* Dynamic traction of lattice-confined platinum atoms into mesoporous carbon matrix for hydrogen evolution reaction. *Sci. Adv.* **4**, eaao6657 (2018).
6. Cheng, X. *et al.* Highly active, stable oxidized platinum clusters as electrocatalysts for the hydrogen evolution reaction. *Energy Environ. Sci.* **10**, 2450-2458 (2017).
7. Ye, S. *et al.* Highly stable single Pt atomic sites anchored on aniline-stacked graphene for hydrogen evolution reaction. *Energy Environ. Sci.* **12**, 1000-1007 (2019).
8. Yan, Q.-Q. *et al.* Reversing the charge transfer between platinum and sulfur-doped carbon support for electrocatalytic hydrogen evolution. *Nat. Commun.* **10**, 4977 (2019).
9. Yu, F.-Y. *et al.* Pt-O bond as an active site superior to Pt^0 in hydrogen evolution reaction. *Nat. Commun.* **11**, 490 (2020).
10. Cheng, N. *et al.* Platinum single-atom and cluster catalysis of the hydrogen evolution reaction. *Nat. Commun.* **7**, 13638 (2016).
11. Yin, X. P. *et al.* Engineering the coordination environment of single-atom platinum anchored on graphdiyne for optimizing electrocatalytic hydrogen evolution. *Angew. Chem. Int. Ed.* **57**, 9382-9386 (2018).
12. Wei, H. *et al.* Ultralow-temperature photochemical synthesis of atomically dispersed Pt catalysts for the hydrogen evolution reaction. *Chem. Sci.* **10**, 2830-2836 (2019).
